# Clear cell sarcoma of soft tissue with plasmacytoid morphology: A rare case report

**DOI:** 10.1097/MD.0000000000031631

**Published:** 2022-11-04

**Authors:** Xu Han, Qingchang Li, En-Hua Wang, Nan Liu

**Affiliations:** a Department of Pathology, the First Affiliated Hospital and College of Basic Medical Sciences, China Medical University, Shenyang, China.

**Keywords:** BRAF, clear cell sarcoma, EWSR1, FISH, melanoma

## Abstract

Clear cell sarcoma of soft tissue (CCSST) is a rare malignant tumor that occurs in the extremities of young adults. CCSST has been documented to have atypical histopathological features, such as epidermotropism or myxoid differentiation, which may set pitfalls in the differential diagnosis. We report a case of CCSST with plasmacytoid morphology which has never been described.

**Patient concerns:**

A 15-year-old male, presented with a 5-cm mass in his left inguinal area.

**Diagnosis:**

Positron emission tomography-computed tomography examination showed nodules in the left groin and the lung, the latter was considered metastasis. A core needle biopsy with the diagnosis of CCSST with plasmacytoid morphology was made according to histology, immunostaining, and molecular analysis.

**Interventions:**

The patient received chemotherapy of doxorubicin and ifosfamide.

**Outcomes:**

The patient failed to respond to the standard chemotherapy and deceased twelve months after diagnosis.

**Lessons:**

This special case of CCSST with plasmacytoid features demonstrated a morphological variation never been documented and may easily lead to misdiagnosis. For such cases, molecular analysis is essential to provide solid evidence for accurate diagnosis.

## 1. Introduction

Clear cell sarcoma (CCS) of soft tissue (CCSST) is a rare subtype of sarcoma comprising approximately 1% of all diagnosed sarcomas.^[1]^ Pathological diagnosis of CCSST is often problematic owing to its immunohistological resemblance to many malignancies such as melanoma, epithelioid malignant peripheral nerve sheath tumor (MPNST), melanotic schwannoma, perivascular epithelioid cell neoplasm (PEComa).^[2]^ Typical CCSST cells can appear with nested or fascicular growth patterns, with pale-staining or clear cytoplasm and basophilic nucleus.^[3,4]^ However, plasmacytoid morphology has never been described in CCSST. Since tumor cells with plasmacytoid morphology can be found in a variety of tumors, this rare morphological variation may lead to misdiagnosis.

Herein, we introduce a case with diagnostic pitfalls due to its plasmacytoid morphology, and a final diagnosis of CCSST was made according to the immunohistochemistry results and *EWSR1* rearrangement verified by fluorescence in situ hybridization.

## 2. Case presentation

A fifteen-year-old male patient presented to the clinic complaining about a growing mass in his left inguinal area. He also recalled nocturnal pain in his left lower extremity started 2 months ago. He was previously healthy but received cryotherapy for a “black mole” on his back 2 years ago. Physical examination revealed a firm subcutaneous mass with fixed mobility. No skin abnormality was found in the surrounding area. Doppler ultrasound showed a 5-centimeter hypoechoic mass. Positron emission tomography-computed tomography examination showed nodules in the left groin and the lung, the latter was considered a “metastasis.” A needle biopsy of the inguinal mass was performed for further diagnosis.

**Figure 1.**
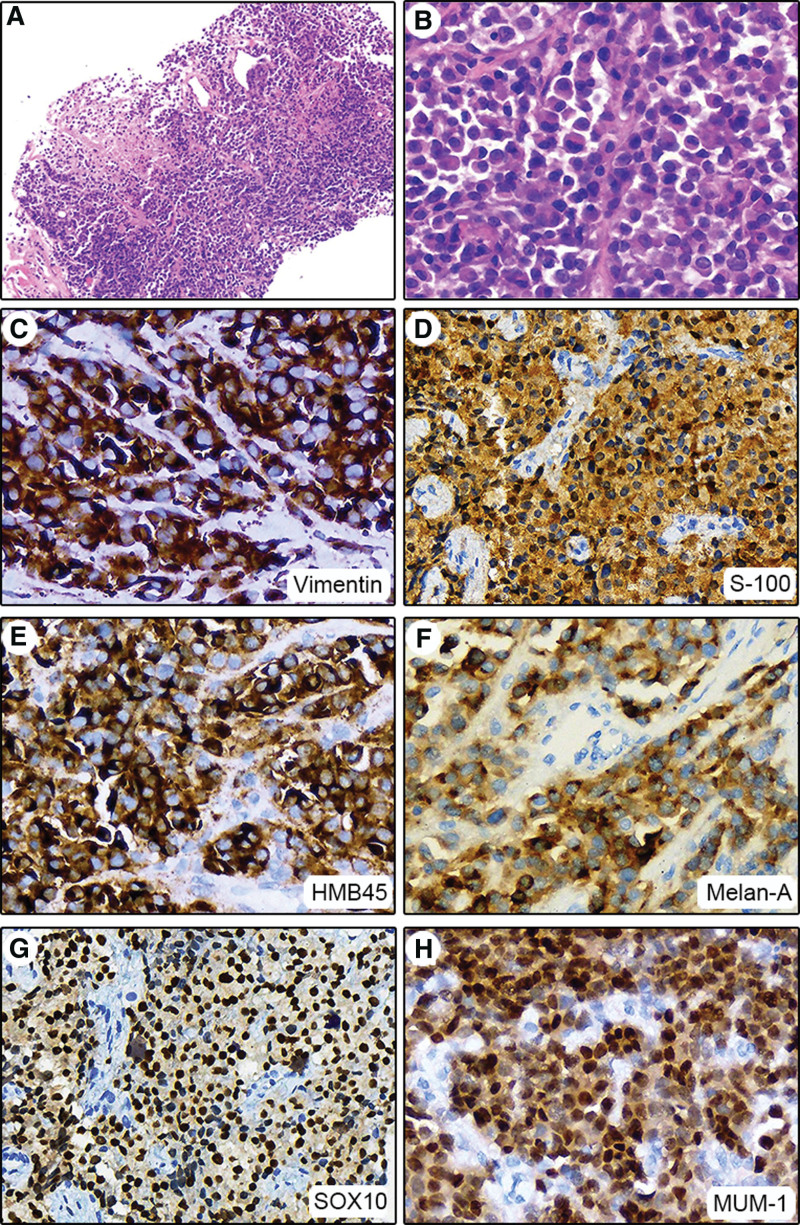
Hematoxylin Eosin stain showed that small compact sheets and nests of tumor cells were divided by fibrous septa, with focal necrosis (A, 40×); higher magnification revealed uniformly sized tumor cells, with eosinophilic cytoplasm and eccentric nuclei. The basophilic nuclei had dense chromatin and prominent nucleoli, resembling plasmocytes (B, 400×). The tumor cells were diffusely expressing vimentin, S-100, HMB45, Melan-A, SOX10, and MUM-1 (C–H, 200×).

In gross, 3 pieces of gray-white tissue ranging from 1.5 to 2 cm were obtained. Microscopically, tumor cells were arranged in small compact sheets and nests, divided into variably sized clusters by fibrous septa, with focal necrosis (Fig. 1A), no melanin pigment was found. Under higher magnification, the tumor cells were uniformly sized, with eosinophilic cytoplasm and eccentric, basophilic nuclei. The nuclei had dense chromatin and prominent nucleoli, resembling plasmocytes (Fig. 1B). Based on the microscopic morphology and medical history, initial diagnostic considerations were plasmacytoid melanoma, extramedullary plasmacytoma, CCSST, malignant PEComa, rhabdomyosarcoma, and myeloid sarcoma. Hence, immunohistochemistry was performed to assist the diagnosis.

**Figure 2.**
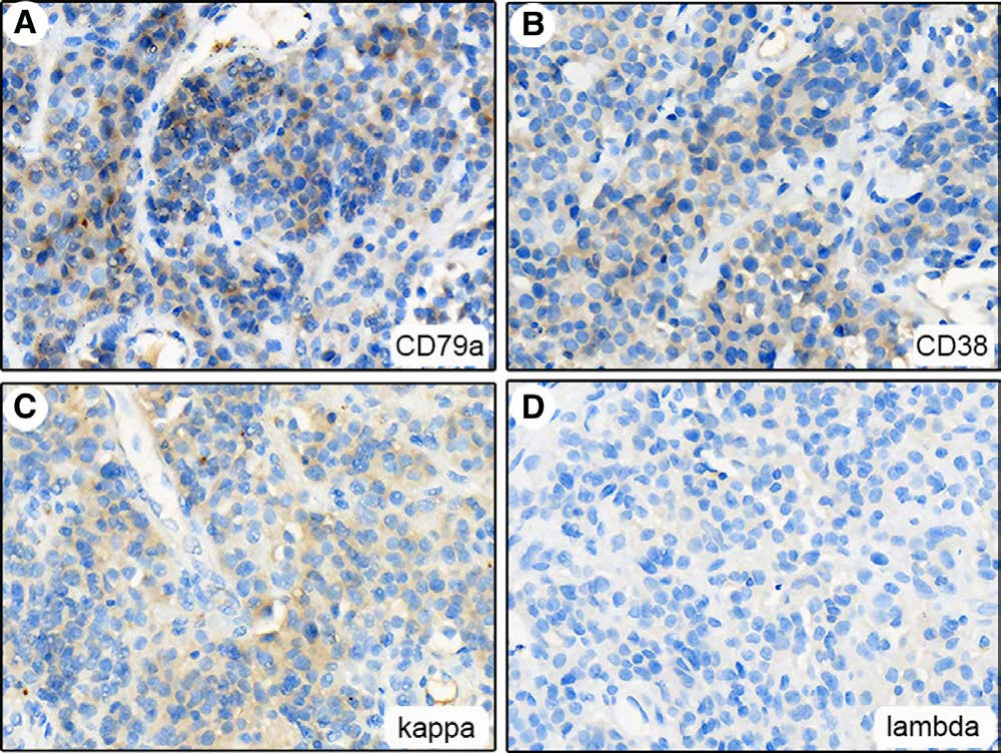
The tumor cells were weakly stained with several B-lymphocyte markers, CD79a, CD38, and kappa (A–C, 200×), while with negative expression of lambda (D, 200×).

**Figure 3.**
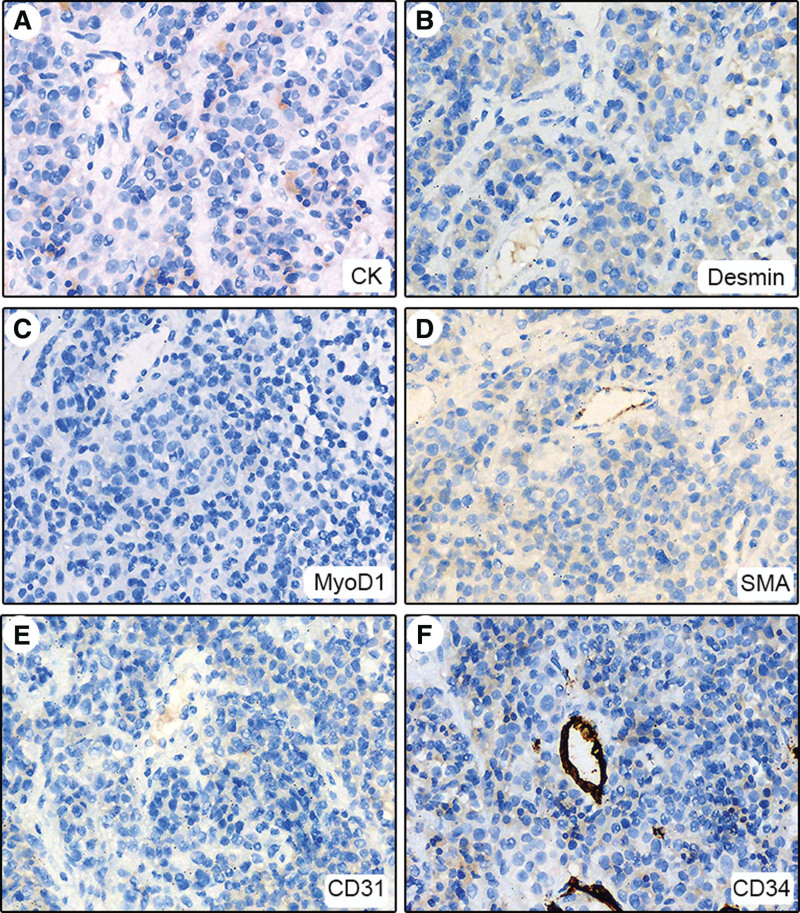
Negative expression of CK, desmin, MyoD1, SMA, CD31, and CD34 in tumor cells (A–F, 200×).

Immunohistochemical studies showed great similarity to the molecular features of melanoma, including strong expression of vimentin, S-100, HMB45, Melan-A, and SOX10 (Fig. 1C–G). Plasma cell marker MUM-1 was strongly positive (Fig. 1H). CD79a, kappa, and CD38 were weakly positive which were considered false positive (Fig. 2A–C); Lambda was negative (Fig. 2D). Others including CD31, CD34, desmin, MyoD1, SMA, and CK were all negative (Fig. 3). Based on these results, extramedullary plasmacytoma, malignant PEComa, rhabdomyosarcoma, and myeloid sarcoma could be excluded.^[5–8]^ Genetic analysis was performed for further differential diagnosis.

**Figure 4.**
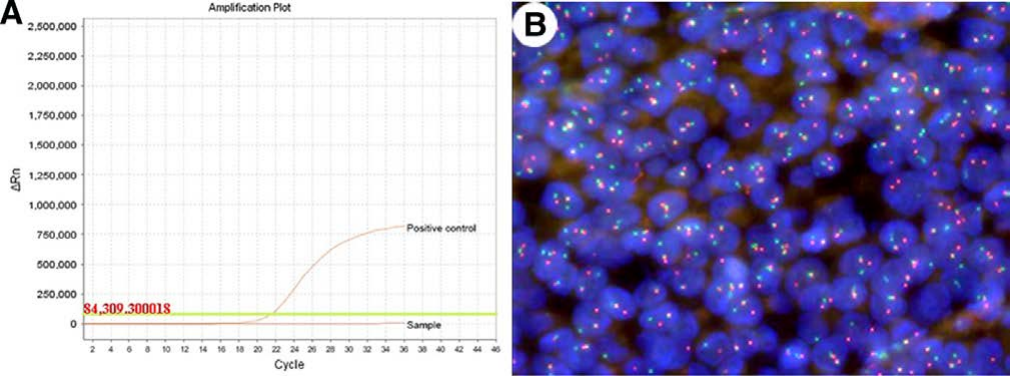
Quantitative polymerase chain reaction showed wild-type *BRAF* gene in this case (A); fluorescence in situ hybridization revealed *EWSR1* translocation in about 85% of tumor cells (splitting signals, B).

The quantitative polymerase chain reaction (qPCR) showed no *BRAFV600* mutation (Fig. 4A). Meanwhile, with *EWSR1* break-apart probe, the fluorescence in situ hybridization analysis revealed *EWSR1* translocation in about 85% of tumor cells (Fig. 4B). Therefore, the pathological diagnosis favored CCS with plasmacytoid morphology. The patient failed to respond to the standard chemotherapy with doxorubicin and ifosfamide and deceased twelve months after diagnosis.

## 3. Discussion and literature review

CCSTA represents a distinct clinicopathologic entity first described by Enzinger in 1965.^[9]^ It is considered a rare malignancy derived from neural crest cells.^[10]^ CCSST primarily occurs in adolescents and young adults with a high propensity for local recurrence, regional lymph node metastases, and distant metastases.^[9,11]^ Although the overall anatomic distribution of CCSST is wide, it has a predilection for the lower extremities, usually intimately associated with fascial structures, and may involve subcutis and dermis by a direct extension.^[12]^ Despite its kinship with malignant melanoma (melanotic differentiation, ultrastructural evidence of melanosomes, and high frequency of metastases), CCSST is genetically different from malignant melanoma or other tumors of neuroectodermal or mesenchymal derivation because of *EWSR1* translocation, a genetic event not frequently seen in melanoma.^[13–15]^

The plasmacytoid morphology fog the diagnosis of this case. Since the patient had a “mole” history, after excluding myogenic tumor through immunohistochemistry, based on the expression of HMB45 and Melan-A, plasmacytoid melanoma became our first consideration. It is generally considered that *BRAF* mutation may contribute to the tumorigenesis and the aggressiveness of malignant melanoma.^[16]^ However, the wild-type *BRAF* gene profile did not fit the early distal (lung) metastasis found in this case. Some melanocytic tumors have been shown to harbor both *EWSR1* amplification and kinase fusions such as *ALK*, *ROS*, *MET*, *BRAF*, or *RET*.^[17]^ Rarely, *BRAF* mutation was also shared by CCS and malignant melanoma but was less frequent in the *EWSR1*-rearranged CCS than in the *EWSR1*-non rearranged CCS.^[18]^ Protsenko et al reported a case of metastatic *EWSR1*-non rearranged and *BRAFV600E*-mutated CCS, which was resistant to conventional chemotherapy of CCS but response well to anti-*BRAF* therapy.^[19]^ Taken together, it seems that *BRAF* mutation is not a diagnostic but a prognostic indicator of CCS.

It is widely accepted that CCSST displays melanocytic differentiation at the light microscopic, ultrastructural, and protein levels, including expression of several conventional melanocytic markers such as S-100, HMB45, MiTF, and Melan-A,^[2,3,14]^ which is also found in this patient. However, the plasmacytoid morphology of CCSST has never been reported. Typical CCS cells range from fusiform to epithelioid and most often contain pale eosinophilic to amphophilic cytoplasm and generally uniform round nuclei with macronucleoli,^[2,3]^ which is inconsistent with this case. Therefore, gene analyses were essential in diagnosing this special case.

It is considered that MUM-1 presents in a wide spectrum of hematolymphoid neoplasms and malignant melanomas.^[20,21]^ Sundram et al^[22]^ reported that 2 cases of CCSST were positive with MUM-1 and S-100 expression, but only 1 demonstrated strong HMB45 staining and neither was positive with Melan-A. Interestingly, diffuse staining of both HMB45 and Melan-A were found in this case, which is also distinct from the previous report. The function of MUM-1 in tumors with melanocytic differentiation, such as melanoma, CCS, and PEComa, is still unclear. However, Ferenczi et al^[23]^ demonstrated differential MUM-1 expression between melanocytic tumors, suggesting MUM-1 as a promising marker in the assistance of differential diagnosis between PEComa and melanoma. They also found MUM-1 positivity in 8 out of 11 CCSST and showed diminished MUM-1 expression intensity in CCSST compared to primary melanomas. Studies are needed to further illustrate the relationship between MUM-1 and conventional melanocytic markers.

The negative CD79a, CD38, kappa, and lambda expression denied possible plasmacytic differentiation of the tumor cells. Since CD138 is not as sensitive and specific as CD38 as plasma cell markers, we preferably used CD38 instead of CD138 to label plasma cells. However, the negative expression of CD38 found in this case unfavored the diagnosis of plasmacytoma in which diffuse membranous CD38 expression is typically found.^[6]^ Moreover, strong, diffuse staining of melanotic markers should not be found in myeloproliferative neoplasms.^[24]^ Therefore, in the current case, we considered that the tumor cells only presented with plasmacytoid morphology in histology, but not plasmacytic differentiation or origin. Nevertheless, in biopsy specimens, plasmacytoid morphology still poses a challenge to diagnosis, because this morphology has never been identified in CCS. In such a situation, the biomarkers of plasma cells and B cells are very helpful for differential diagnosis.

Thus far, no compelling immunohistochemical markers have been found that reliably differentiate CCSST and melanoma. A genetic study for reciprocal *EWSR1* translocation is required for definitive diagnosis. It has subsequently become clear that > 90% of CCS cases are associated with this translocation.^[2,3,14]^ Rearrangements involving the *EWSR1* gene have been implicated in many sarcomas.^[25]^ Emerging evidence suggests fusion protein *EWSR1/ATF1* or *EWSR1/CREB1* expression can drive sarcomagenesis. *EWS/ATF1* expression inhibits oncogene-induced senescence in sarcoma cells.^[26]^ In the presence of SOX10, *EWS/ATF1* fusion protein can bind to and activate MiTF, which results in melanocytic phenotype expression and growth/survival of CCS cells.^[27]^ Besides, a genomic study in the mice CCS model indicated *MiTF* amplification contributed to *EWS/ATF1*-driven tumorigenesis.^[28]^ These findings explain the rapid progression and early metastasis seen in this patient.

It is worth mentioning that *MYC* amplification was also found in mice CCS model and could not only potentiate *EWSR1-ATF1*-driven sarcomagenesis but also alter tumor histomorphology, with variants of myxoid and nested phenotypes.^[28]^ A *MiTF-CREM* fusion has recently been reported in a clear cell neoplasm with melanocytic differentiation, which was considered a novel entity of clear cell tumor with similarity to CCS.^[29]^ Identification and analysis of additional CCS-like cases should be conducted to clarify the nosologic status and the biologic potential of clear cell neoplasms.

## 4. Conclusion

We reported a special CCS with plasmacytoid features. This rare variation may easily lead to misdiagnosis. Since histomorphologic examination may not always provide an unambiguous diagnosis, genetic examination to make the distinction is essential. The diagnosing process of this case once again prompted the necessity of gene analysis in the diagnosis of soft tissue tumors.

## Author contributions

All authors made a significant contribution to the work reported, whether that is in the conception, study design, execution, acquisition of data, analysis, and interpretation, or in all these areas; took part in drafting, revising, or critically reviewing the article; gave final approval of the version to be published; have agreed on the journal to which the article has been submitted; and agree to be accountable for all aspects of the work.

**Methodology**: Nan Liu.

**Project administration**: Qingchang Li.

**Supervision:** En-Hua Wang.

**Writing** – original draft: Xu Han.

**Writing** – review & editing: Nan Liu.
